# Fermentation of Foods and Beverages as a Tool for Increasing Availability of Bioactive Compounds. Focus on Short-Chain Fatty Acids

**DOI:** 10.3390/foods9080999

**Published:** 2020-07-25

**Authors:** Giuseppe Annunziata, Angela Arnone, Roberto Ciampaglia, Gian Carlo Tenore, Ettore Novellino

**Affiliations:** 1 Department of Pharmacy, University of Naples Federico II, Via Domenico Montesano 49, 80131 Naples, Italy; giuseppe.annunziata@unina.it (G.A.); roberto.ciampaglia@unina.it (R.C.); ettore.novellino@unina.it (E.N.); 2 Dipartimento di Medicina Clinica e Chirurgia, Unit of Endocrinology, Federico II University Medical School of Naples, Via Sergio Pansini 5, 80131 Naples, Italy; angela.arnone15@gmail.com

**Keywords:** short-chain fatty acids, microorganisms, fermentation, nutraceutical, functional foods

## Abstract

Emerging evidence suggests that fermentation, historically used for the preservation of perishable foods, may be considered as a useful tool for increasing the nutritional value of fermented products, in terms of increases in bioactive compound content, including short-chain fatty acids (SCFAs), as bacteria end-products, whose beneficial effects on human health are well-established. The purpose of the present manuscript is to summarize studies in this field, providing evidence about this novel potential of fermentation. A limited number of studies directly investigated the increased SCFA levels in fermented foods. All studies, however, agree in confirming that levels of SCFAs in fermented products are higher than in unfermented products, recognizing the key role played by the microorganisms in metabolizing food matrices, producing and releasing bioactive substances. According to the available literature, fermentation might be taken into account by the food industry as a *natural* strategy with no environmental impacts to produce functional foods and beverages with a higher nutritional value and health-promoting compounds.

## 1. Introduction

Fermentation has been historically used as a strategy to increase the shelf-life of perishable foods [1]. Over time, boththe production and consumption of fermented foods and beverages have strongly increased, due to their peculiar and appreciated taste, and their recognized health benefits. Over the last few decades, scientific research has focused attention on the beneficial properties of fermented foods and beverages, and their impact on human health [2]. Besides their well-established role on gastrointestinal tract, there is increasing evidence demonstrating that this class of products is able to ameliorate several metabolic outcomes, including glycemia, lipidemia, and oxidative stress, as shown in animal-based in vivo studies [3,4].

**Figure 1:**
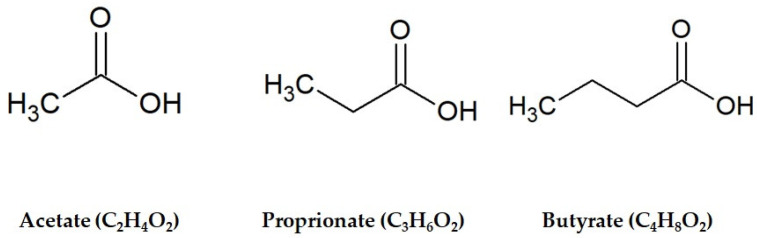
Chemical structures of the main short-chain fatty acids.

Notably, fermentation has been indicated as a tool to enhance the nutritional value of foods and beverages, in terms of both increased bioavailability of bioactive compounds (i.e., polyphenols) and production of health-promoting end-products. Among the latter, short-chain fatty acids (SCFAs) are emerging as some of the most studied compounds in the last decade, due to their proven beneficial impact on human health. SCFAs are small organic monocarboxylic acids with different chain lengths, ranging from two to six carbon atoms. They are prevalently produced by gut microbiota as end-products of fermentation of dietary polysaccharides, including fiber and resistant starch. The most representative SCFAs are acetic acid (C2), propionic acid (C3) and butyric acid (C4) (Figure 1), produced in a molar rate of about 60:20:20, respectively [5].

Evidence shows that SCFAs may be efficiently involved inglycemic control [6]. The main putative mechanism would involve G protein-coupled receptors (GPCRs) [7], whose activation by SCFAs leads to secretion of enterohormones, including glucagon-like peptide-1 (GLP-1) and peptide tyrosine–tyrosine (PYY), resulting in improved glucose tolerance and increased insulin release [8,9]. Interestingly, an animal-based study reported that acetate, produced by colonic fermentation, was able to cross the blood–brain barrier and act as an appetite suppressant in hypothalamus [10]. Overall, this evidence suggests that SCFAs might play a relevant role in the management of metabolic diseases, including obesity and diabetes, where the control of food intake is crucial. Moreover, propionate has been shown to reduce both liponeogenesis and cholesterol synthesis [11]. Furthermore, it has been demonstrated that an increased influx of SCFAs into the liver results in decreased levels of angiopoietin-like protein 4 (ANGPTL4), a target gene of peroxisome proliferator-activated receptors (PPARs) [12]. These specific transcription factors regulate the expression of genes [13] involved in various cell functions, including differentiation, the metabolism of carbohydrates, protein and lipids [14], and tumorigenesis [15]. In addition to these metabolic effects, studies demonstrated the positive influence of SFCAs on intestinal functions. In particular, it has been reported that SCFAs are used as an energy source by colonic epithelial cells [16], contributing to the maintenance of colonic mucosa integrity [17]. These data suggest the role of SCFAs in both the prevention and treatment of intestinal diseases [18]. Interestingly, SCFAs, mainly butyrate, have been demonstrated to possess an anticancer potential, due to their ability to regulate proliferation, differentiation and cell apoptosis, via inhibition of histone-D-acetylase [19], and anti-inflammatory activity, via suppression of pro-inflammatory pathways [20]. These mechanisms of action would, at the base, decrease the production of pro-inflammatory cytokines, including tumor necrosis factor (TNF)α, interleukin (Il)-1β, -6 [21], -12 [22], and up-regulate anti-inflammatory cytokines, including IL-10 [21].

Despite this promising evidence, most of the studies investigating the beneficial effects of SCFAs on human health focused on the role played by endogenous SCFAs, produced in the lower intestine by microbiota fermentation. On the other hand, limited literature is available on the biological effects of exogenous SCFAs occurring in fermented products.

The purpose of the present mini review is to summarize the available literature of the last 5 years, in order to elucidate the potential role of fermented products as a source of SCFAs. In particular, a literature search was conducted by consulting official scientific databases, including PubMed (http://www.ncbi.nlm.nih.gov/pubmed) and Science Direct (http://www.sciencedirect.com). Articles in English were downloaded using specific keywords (“fermented foods”, “fermented beverages”, “short-chain fatty acids”, “butyrate”, “acetate”, “propionate”) and their combinations.

## 2. Increased Bioaccessibility and Bioavailability of Bioactive Compounds Occurring in Fermented Products: A Prime Example of the Usefulness of Fermentation

Emerging evidence reports that, during fermentation, there is a marked enhancement of the nutritional value of final products [23,24], in terms of increased bioaccessibility and bioavailability of bioactive compounds. As an example, several studies have focused on the increase of polyphenol content in fermented foods.

In fruit and vegetables, polyphenols mainly bind carbohydrate residues in the plant cell wall [25], resulting in variable intestinal absorption. In this context, microorganism fermentation potentially acts by: (i) releasing bioactive compounds from fiber by degrading it, and then (ii) metabolizing them, releasing smaller molecules or their metabolites, which are more efficiently absorbed at the intestinal level. This is probably due to the ability of microorganisms to express amylase, β-glucosidase, decarboxylase, phenolic acid decarboxylases, esterase, phenol reductase, tannase and glucoamylase, which have been described for their pivotal role played in enhancing polyphenol levels in fermented foods [26,27]. The expression of these enzymes is strain-specific [26].

The activity of microorganisms on food matricesduring fermentation is, thus, crucial to obtain high nutritional value of the final products. Previous authors have reported the increase in the antioxidant activity in fermented products compared with non-fermented ones. These observed results might be explained by the above mentioned activities of microorganisms.

Further studies investigated the role of intestinal microbiota in increasing both polyphenol bioaccessibility and antioxidant activity, providing evidence regarding the ability of bacteria to metabolize non-absorbed polyphenols that reach the colon [25,28,29,30,31]. This evidence concerns not only polyphenol-rich foods but also nutraceutical products, as demonstrated in a recent study in which acid-resistant capsules containing tea polyphenolic extract were submitted to a simulated gastrointestinal digestion protocol, including both upper and lower intestinal digestion steps. Following the colonic digestion, researchers noted a significant increase in both polyphenol levels and antioxidant activity, suggesting that gut microbiota is able to further metabolize polyphenols, making them more bioavailable and active [31].

Overall, this evidence suggests that microorganisms used for fermentation are able to metabolize the food matrix and release bioactive compounds. This, in turn, enhances the nutritional value of final fermented products. In this sense, as aforementioned, both bioaccessibility and bioavailability of polyphenols (naturally bound to fibers) are increased in fermented products. These aspects indicate fermentation as a useful strategy for the production of functional foods.

## 3. Increased Levels of SCFAs in Fermented Foods and Beverages

A limited number of studies investigated the amount of SCFAs in fermented products. Nevertheless, authors generally agree that fermentation significantly increases the levels of this class of bioactive compounds (Table 1). Different raw materials have been studied, including milk [32,33,34], beer wort [35], fruit [36] and vegetables [2], and several bacteria strains have been used for fermentation.

Yoghurt is undoubtedly one of the most common fermented products, obtained from the fermentation of milk by specific bacterial strains capable of fermenting it by breaking down molecular bonds with sugars, mainly lactose, and producing acids (mainly lactic acid), with the consequent coagulation of proteins which thicken the matrix [39,40,41]. Besides the well-established beneficial effects of yoghurt consumption on human health, a limited number of studies focused on the evaluation of SCFA content in fermented milk. Jia et al. [33] demonstrated that in goat milk fermented with *Lactobacillus rhamnosus GG*, the amount of total SCFAs was significantly higher than that in non-fermented goat milk (*p* < 0.05). On the other hand, the levels of long-chain fatty acids (LCFAs) progressively decreased, suggesting that lipoprotein lipase might be responsible for hydrolyses of LCFAs, leading to the production of both SCFAs and medium-chain fatty acids (MCFAs). In the same study, goat milk was also fermented with traditional yoghurt starter cultures, including *Lactobacillus delbrueckii ssp. bulgaricus* and *Streptococcus thermophilus*. In these samples, the trend of fatty acid production was inverted, with an increase in LCFAs, and a decrease in SCFA and MCFA levels. According to the authors, the addition of mixed starter cultures may result in a partial inhibition of lipoprotein lipase activity [33], suggesting that the use of a specific bacterial strain, rather than another, may be a useful tool to increase the nutritional value of fermented products. Another research group analyzed different samples of human milk, commercial pure cow milk, infant formula and fermented cow milk, finding highest amount of SCFAs in fermented cow milk [34]. In line with this evidence, a further study demonstrated that the fermentation of skimmed milk with *Lactobacilli* or *Bifidobacteria* increased SCFA production with variable trends, based on the type of added prebiotics, including β-glucan, inulin and hi-maize. Generally, after 24 h fermentation, higher amounts of SCFAs (acetate 2.72 mM, propionate 0.92 mM and butyrate 0.41 mM) were found in samples fermented with *Bifidobacteriumanimalis subsp. Lactis* and inulin as prebiotic, suggesting a strain- and substrate-dependent relationship [32]. It is clear that various microbe strains have different metabolic pathways, and this evidence may guide research toward the choice of the best combination of raw material and microorganisms, in order to optimize the production of SCFAs in the final fermented products. In line with this evidence, an elegant study compared both rate and profile of SCFA production during fermentation of rice fiber with different bacterial strains, including *Lactobacillus rhamnosus, Lactobacillus acidophilus* and *Bifidobacteriumlongum* [42]. In general, the amount of SCFAs was higher when samples were treated with an inter-genus than intra-genus combination. In addition, acetate was the most produced metabolite, and butyrate the least. Despite the well-known metabolic pathways of *Bifidobacteria*, including the Wood–Ljungdahl pathway, the authors suggested that the fructose-6-phosphoketolase-involving pathway, also known as “bifid shunt”, was a further mechanism for carbohydrate metabolism, resulting in SCFA production. In contrast, *Lactobacilli* mainly produced acetate through the transketolase pathway, in a significantly lower rate than *Bifidobacteria*. The combination of these two genera, thus, leads to a higher production of SCFAs [42].

Utoiu et al. [38] performed a laboratory experiment, proving that fermentation of Kombucha beverage with pollen led to a significant increase in SCFAs. In particular, they used a mixture of microorganisms called *symbiotic culture of bacteria and yeast (SCOBY*), which included lactic acid bacteria, acetobacteria, and yeasts from various genera. Both laboratory-level and large-scale experiments were performed with different fermentation duration periods (0–17 days and 18 days, respectively). Pollen addition contributed to a significant enhancement in SCFA production in both experiments. In particular, SCFAs progressively increased during fermentation days (in laboratory level: acetate from 0.415 ± 0.005 g/L to 3.51 ± 0.11 g/L, propionate from 0.095 ± 0.012 g/L to 0.56 ± 0.041 g/L, butyrate from 0.12 ± 0.038 g/L to 1.78 ± 0.054 g/L), reaching their highest amount in 18 days (large-scale: acetate 19.56 ± 0.18 g/L, propionate 0.66 ± 0.037 g/L, butyrate 1.92 ± 0.033 g/L) [38]. The large microbial diversity in SCOBY, and, in particular, the high content of lactic acid bacteria, might explain the high SCFA production. Similarly, it has been noted that butyrate levels significantly increased in short-time tea infusion fermented with lactic acid bacteria (including *Lactobacillus bulgaricus*, *L. acidophilus*, *L. rhamnosus, Lactobacillus plantarum*), in a time-dependent manner. In particular, the highest concentrations of butyrate were detected after (i) 15-min fermentation with *L. rhamnosus* (1.332 ± 0.065 µg/g), (ii) 30-min fermentation in samples treated with *L. bulgaricus* and *L. plantarum* (3.642 ± 0.058 µg/g and 2.157 ± 0.364 µg/g, respectively), and (iii) 120-min fermentation with *L. acidophilus* (2.479 ± 0.137 µg/g) [37]. The fermentation of fiber occurring in herb samples might be considered as the main mechanism for the observed increase in SCFA levels, despite the short fermentation time (from 7 to 180 min). In non-fermented samples, butyrate was not detected.

Besides the well-known Kombucha, many other fermented beverages are traditionally produced and consumed worldwide, mainly alcoholic beverages. Among these, beer holds a prominent place. It has been demonstrated that during fermentation, SCFA concentrations significantly increase, from 1.2–2.2 mg/L to 2.3–8.1 mg/L. It seems that the initial steps during the brewing process are also able to influence the amount of SCFAs in the final product. In particular, when infusion mashing is used, the total amount of SCFAs, mainly, butyrate, is higher when compared with decoction [35].

Due to their high content in sugar and non-digestible carbohydrates, fruits may be considered one of the best raw material for fermented products [43]. It has been reported that the fermentation of raw guava fruits with *L. plantarum* significantly increase the content of butyrate compared to the unfermented control (unfermented: 1.30 ± 0.39 ng/100 mL; fermented: 17.85 ± 0.68 ng/100 mL) [36]. This would suggest that fruit carbohydrate may represent a useful substrate for bacterial fermentation, leading to the increased production of bioactive end-products, including SCFAs. Similar to fruits, vegetables can also be considered a good raw material for fermented products. Recently, a study reported a significant increase in SCFAs in *L. rhamnosus GG*-fermented carrot juice, compared with non-fermented ones (unfermented carrot juice: acetate 0.16 ± 0.02 mg/mL, propionate 0.51 ± 0.07 mg/mL, butyrate 0.64 ± 0.11 mg/mL; fermented carrot juice: acetate 0.42 ± 0.05 mg/mL, propionate 0.72 ± 0.09 mg/mL, butyrate 0.995 ± 0.09 mg/mL;*p*<0.05). Additionallyin this case, the fermentation of dietary fiber was considered as the main mechanism for the increased production of SCFAs [2].

## 4. Conclusions and Future Perspectives

**Figure 2:**
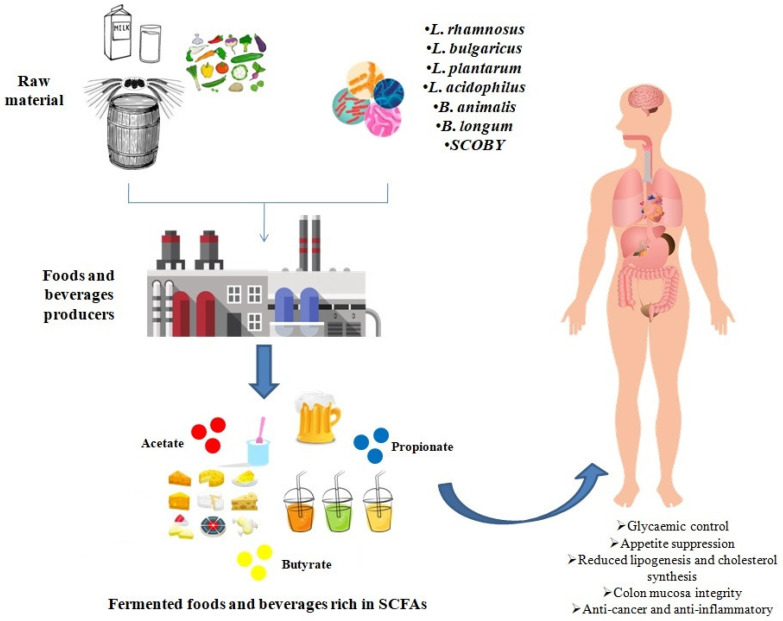
Fermentation with different microorganisms might be used by food industry to produce functional foods and beverages enriched in short-chain fatty acids that can exert several beneficial effects on human health.

In this manuscript, we reported evidence from various studies monitoring the levels of SCFAs in various fermented foods and beverages, reaching the conclusion that fermentation is a useful strategy to increase the levels of SCFAs in fermented foods and beverages. More specifically, microorganisms produce SCFAs as end-products via the metabolism of food components, such as fiber and other carbohydrates. In this sense, fermentation should be taken into account by the food industry as a possible process with neither additional impact on the environment, nor further food processing for the production of functional foods and beverages (Figure 2).

The available studies herein reported, however, led to individuate various concerns. Firstly, different microorganisms and prebiotics were used in these studies. This makes establishing the best criteria for the fermentation process in order to optimize SCFAs production difficult. Then, although promising, these studies are limited in number and, to our knowledge, based exclusively on in vitro evidence. No studies were conducted in humans, chronically administered with fermented products in order to test the effects of exogenous SCFAs. These are weak points hindering the use of fermentation by the food industry for the production of functional foods. In this sense, further studies are needed to individuate the best combination of raw materials, microorganisms, prebiotics, and fermentation times. In parallel, clinical trials, aimed to confirm the health-promoting effects of SCFAs, administered with fermented foods and beverages, should be performed.

## Figures and Tables

**Table 1:** Studies reporting the increased levels of short-chain fatty acids (SCFAs) in fermented foods.

Raw Material	Microorganism	Main Results	Reference
Carrot juice	*Lactobacillus rhamnosus*	➢ Non-fermented: acetic acid 0.16 ± 0.02 mg/mL; propionic acid 0.51 ± 0.07 mg/mL; butyric acid 0.64 ± 0.11 mg/mL ➢ Fermented: acetic acid 0.42 ± 0.05 mg/mL; propionic acid 0.72 ± 0.09 mg/mL; butyric acid 0.95 ± 0.09 mg/mL	[2]
Skim milk	*Lactobacilli* and *Bifidobacteria*	➢ Skim milk supplemented with 5% *w/v* inulin using *B. animalis* subsp. *lactis*: acetic acid 2.72 mM; propionic acid 0.92 mM; butyric acid 0.41 mM ➢ Skim milk supplemented with 5% *w/v* hi-maize using *B. animalis* subsp. *lactis*: acetic acid 2.11 mM; propionic acid 0.68 mM; butyric acid 0.24 mM ➢ Skim milk supplemented with 5% *w/v* inulin using *L. rhamnosus GG*: acetic acid 2.56 mM; propionic acid 0.85 mM; butyric acid 0.34 mM ➢ Skim milk supplemented with 5% *w/v* hi-maize using *L. rhamnosus GG*: acetic acid 2.58 mM; propionic acid 0.70 mM; butyric acid 0.25 mM	[32]
Goat milk	*Lactobacillusrhamnosus GG*	SCFAs: C4 +5.63%; C6 +5.86%; C8 +5.15%; C10 + 2.33%, total SCFAs +3.35%, compared to non-fermented goat milk	[33]
Beer wort	*Yeast*	➢ Non-fermented: total SCFAs (butyric, isobutyric, isovaleric, caprylic and caproic acid) 1.2–2.2 mg/L ➢ Fermented: total SCFAs (butyric, isobutyric, isovaleric, caprylic and caproic acid) 2.3–8.1 mg/L	[35]
Guava fruit	*Lactobacillus plantarum*	➢ Non-fermented: butyrate 1.30 ± 0.39 ng/100 mL; caproate 1.00 ± 0.34 ng/100 mL; caprylate 2.40 ± 0.31 ng/100 mL; caprate 1.58 ± 0.27 ng/100 mL; laurate 9.67 ± 0.36 ng/100 mL ➢ Fermented: butyrate 17.85 ± 0.68 ng/100 mL; caproate 62.03 ± 0.55 ng/100 mL; caprylate 34.93 ± 0.62 ng/100 mL; caprate 6.97 ± 0.52 ng/100 mL; laurate 17.97 ± 0.51 ng/100 mL	[36]
Tea	*Lactobacillus plantarum, Lactobacillus acidophilus, Lactobacillus rhamnosus* and *Lactobacillus bulgaricus*	➢ Non-fermented: butyric acid ND ➢ Fermented with *L. plantarum*—7-min fermentation butyric acid 1.494 ± 3.04 µg/g; 15-min fermentation butyric acid 1.693 ± 0.500 µg/g; 30-min fermentation butyric acid 2.157 ± 0.364 µg/g; 45-min fermentation butyric acid 1.385 ± 0.027 µg/g; 60-min fermentation butyric acid 1.459 ± 0.070 µg/g; 90-min fermentation butyric acid 1.558 ± 0.036 µg/g; 120-min fermentation butyric acid 1.367 ± 0.002 µg/g; 180-min fermentation butyric acid 1.277 ± 0.024 µg/g ➢ Fermented with *L. acidophilus*—7-min fermentation butyric acid ND; 15-min fermentation butyric acid 1.846 ± 0.0.763 µg/g; 30-min fermentation butyric acid 1.700 ± 0.181 µg/g; 45-min fermentation butyric acid 1.421 ± 0.290 µg/g; 60-min fermentation butyric acid 1.475 ± 0.634 µg/g; 90-min fermentation butyric acid 1.995 ± 0.0.106 µg/g; 120-min fermentation butyric acid 2.479 ± 0.137 µg/g; 180-min fermentation butyric acid 1.561 ± 0.033 µg/g ➢ Fermented with *L. rhamnosus*—7-min fermentation butyric acid 1.195 ± 0.407 µg/g; 15-min fermentation butyric acid 1.332 ± 0.0.065 µg/g; 30-min fermentation butyric acid 0.889 ± 0.0.023 µg/g; 45-min fermentation butyric acid 0.990 ± 0.050 µg/g; 60-min fermentation butyric acid 1.014 ± 0.019 µg/g; 90-min fermentation butyric acid 0.864 ± 0.028 µg/g; 120-min fermentation butyric acid 1.007 ± 0.005 µg/g; 180-min fermentation butyric acid 1.014 ± 0.053 µg/g ➢ Fermented with *L. bulgaricus—*7-min fermentation butyric acid 2.037 ± 0.005 µg/g; 15-min fermentation butyric acid 2.312 ± 0.087 µg/g; 30-min fermentation butyric acid 3.642 ± 0.058 µg/g; 45-min fermentation butyric acid 2.848 ± 0.049 µg/g; 60-min fermentation butyric acid 2.772 ± 0.066 µg/g; 90-min fermentation butyric acid 2.936 ± 0.036 µg/g; 120-min fermentation butyric acid 2.727 ± 0.126 µg/g; 180-min fermentation butyric acid 2.2880 ± 0.031 µg/g	[37]
Kombucha with pollen	*SCOBY*	➢ Kombucha—non-fermented: acetic acid 0.375 ± 0.005 g/L; propionic acid ND; butyrate ND ➢ Kombucha—fermented: acetic acid 4.46 ± 0.025 g/L; propionic acid 0.24 ± 0.016 g/L; butyrate 0.30 ± 0.021 g/L ➢ Kombucha+pollen—non-fermented: acetic acid 0.415 ± 0.005 g/L; propionic acid 0.095 ± 0.012 g/L; butyrate 0.12 ± 0.038 g/L ➢ Kombucha+pollen—fermented: acetic acid 3.51 ± 0.11 g/L; propionic acid 0.56 ± 0.041 g/L; butyrate 1.78 ± 0.054 g/L	[38]
